# Anisotropic Reaction Properties for Different HMX/HTPB Composites: A Theoretical Study of Shock Decomposition

**DOI:** 10.3390/molecules27092787

**Published:** 2022-04-27

**Authors:** Zheng-Hua He, Yao-Yao Huang, Guang-Fu Ji, Jun Chen, Qiang Wu

**Affiliations:** 1 National Key Laboratory of Shock Wave and Detonation Physics, Institute of Fluid Physics, China Academy of Engineering Physics, Mianyang 621900, China; herary-hezhh@caep.cn (Z.-H.H.); huangyaoyao18@gscaep.ac.cn (Y.-Y.H.); cyfjkf@126.com (G.-F.J.); 2 National Key Laboratory of Computational Physics, Institute of Applied Physics and Computational Mathematics, Beijing 100088, China

**Keywords:** HMX/HTPB, molecular dynamics, shock decomposition, reaction mechanism

## Abstract

Plastic-bonded explosives (PBXs) consisting of explosive grains and a polymer binder are commonly synthesized to improve mechanical properties and reduce sensitivity, but their intrinsic chemical behaviors while subjected to stress are not sufficiently understood yet. Here, we construct three composites of β-HMX bonded with the HTPB binder to investigate the reaction characteristics under shock loading using the quantum-based molecular dynamics method. Six typical interactions between HMX and HTPB molecules are detected when the system is subjected to pressure. Although the initial electron structure is modified by the impurity states from HTPB, the metallization process for HMX does not significantly change. The shock decompositions of HMX/HTPB along the (100) and (010) surface are initiated by molecular ring dissociation and hydrogen transfer. The initial oxidations of C and H within HTPB possess advantages. As for the (001) surface, the dissociation is started with alkyl dehydrogenation oxidation, and a stronger hydrogen transfer from HTPB to HMX is detected during the following process. Furthermore, considerable fragment aggregation is observed, which mainly derives from the formation of new C−C and C−N bonds under high pressure. The effect of cluster evolution on the progression of the following reaction is further studied by analyzing the bonded structure and displacement rate.

## 1. Introduction

Octahydro-1,3,5,7-tetranitro-1,3,5,7-tetrazocine (HMX), as one of the most popular high performance explosives (HEs) used in the weapons field, possesses many outstanding characteristics [1,2,3,4] of high density, ideal detonation velocity, and pressure as well as considerable thermal stability. However, it also has an drawback of high sensitivity when subjected to mechanical friction and impact. How to reduce its sensitivity and largely keep its detonation performance is still a challenge.

Generally, two mainly strategies can be carried out to improve the mechanical security of explosives, which are the improvement of its crystallization craft [5,6] and development of a new complex formula [7,8,9]. Among these strategies, plastic-bonded explosives (PBXs) are the most extensively studied and are practically applied in military fields, ascribing to their good mechanical property and easy processing. This kind of energetic composite commonly consists of grain explosives, polymer binder, and a few additives. Picking a suitable binder to enhance compatibility between different components is the key point for PBXs synthesis. Hydroxyl-terminated poly-butadiene (HTPB) [10,11] is one of the most common binders used for composite explosives and solid propellants. The microscopic structure and interaction mechanism of HTPB with grain explosives, similar to RDX, HMX, have been systematically investigated [12,13,14,15]. The typical hydrogen bond interactions (between hydroxyl-H and nitro-O, hydroxyl-O and alkyl-H of HMX, HTPB alkyl-H and nitro-O) and mechanical characteristics of HTPB-bonded PBXs are revealed in previous theoretical works [13].

Meanwhile, the chemical behaviors subjected to thermal loading are also widely explored [16,17,18,19,20,21,22]. Abd-Elghany et al. [18] studied the reaction activity of an HMX/HTPB compound using the Vacuum Stability Test technique and proposed that HTPB binder could reduce the thermal stability of HMX, causing a lower reaction energy barrier. Singh et al. [19] compared the thermal dissociation of RDX and RDX/HTPB composites, and suggested that the additional HTPB molecules were beneficial to condensed- phase explosive decomposition. Wu and coworkers [16] explored the thermal decomposition process of the HMX/HTPB composite based on molecular dynamics simulation and uncovered the microscopic interaction mechanism of HTPB on HMX decomposition. Furthermore, the shock behaviors had also been investigated for these kinds of composite, such as when Dhiman et al. [23] analyzed the stress wave propagation within HMX/HTPB, when Dlott et al. [24] revealed the hot spot formation in PBX explosives under shock loading, and when Olokun et al. [25] reported the effect of microstructure on impact induced temperature rise in HMX/HTPB. However, the microscopic structure response and reaction properties are seldom investigated, and the intrinsic interaction mechanism between binder and explosive molecules under shock loading still keeps its obscuration.

In this study, we construct three HMX/HTPB composites to investigate their shock reaction behaviors. The microscopic interaction mechanism and electron properties subjected to shockwaves are analyzed. The anisotropic shock decomposition behaviors are systematically studied based on the analysis of population evolutions of key intermediate products and primary chemical bonds. The effects of HTPB binder on HMX shock dissociation are proposed and compared with thermal decomposition. The reaction activity change relative with carbon aggregation is revealed. 

## 2. Computational Methods

The structure optimization and shock decomposition of HMX/HTPB composites are performed using the density functional tight-binding method (DFTB) [26] combined with the multiscale shock technique (MSST) [27] implemented in CP2K code [28]. The self-consistent charge (SCC) manner is employed for description of total energies, atomic forces, and charge transfer. This coupled calculation method has been widely used to investigate the microscopic structure and reaction properties of common energetic materials [29,30,31,32,33], such as TATB, HMX, NM, which always produces consistent results with the relevant experiment and other density functional theory (DFT) calculations. The electron features of HMX/HTPB composites are explored by analyzing the density of states (DOS) based on DFT calculations. The norm-conserving Goedecker, Teter, Hutter (GTH) [34] pseudo-potentials and PBE [35] exchange-correlation functions are used to describe the core–electron interaction.

**Figure 1:**
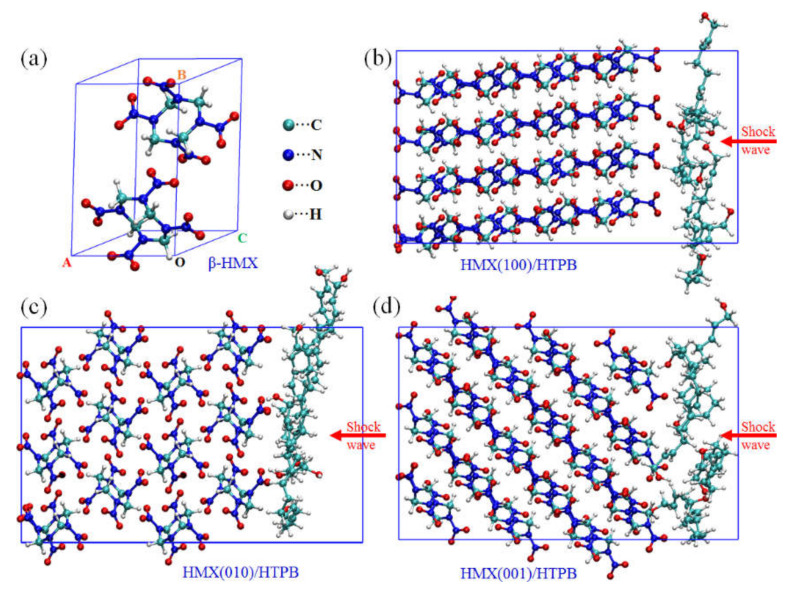
Initial models for HMX/HTPB compositions under shock loading: (**a**) β-HMX crystal, (**b**) HMX(100)/HTPB, (**c**) HMX(010)/HTPB, (**d**) HMX(001)/HTPB.

The initial structure of β-HMX is determined by X-ray experiment [36], which is a typical monoclinic configuration (see Figure 1a), with crystal lattice parameters of 6.54, 11.05, 8.70 Å; and 90°, 124.30°, 90°, respectively. The geometry optimization for the unit cell is first carried out to obtain a stable structure using the DFTB method. Then, three supercells with different surfaces (4×2×2-(100), 3×2×3-(010), and 3×2×3-(001)) of a β-HMX crystal are constructed. Four HTPB molecules with different polymerization degrees are adsorbed on these surfaces by annealing the strategy to form the initial HMX/HTPB composites (see Figure 1b–d), with a mass fraction of 8.6%. More details about the model construction is illustrated in our previous work [16]. A sequential molecular dynamics simulation is performed with time steps of 0.1 fs and SCF convergence of 10^−6^ au. The composites are all equilibrated at 300 K for 1 ps with an *NVT* ensemble. After that, a 9 km·s^−1^ shock wave is perpendicularly loaded onto the surface to guarantee a fast reaction process, which also corresponds to a stable detonation speed of β-HMX. The fictitious cell mass is set to 3 × 10^7^, 4.5 × 10^7^ and 4 × 10^7^ au for composites of HMX(100)/HTPB, HMX(010)/HTPB, HMX(001)/HTPB, respectively. All the simulations are carried out for 4 ps. The MD trajectories are analyzed stepwise with our postprocessing procedure, and the stable molecular components are identified based on chemical bond length and lifetime criteria. [29] If two atoms keep their interaction distance within a critical value of *R*_c_ for more than 10 fs (*R*_c_ is determined by the mulliken bond order as proposed in a previous work [30]), they are defined as bonded pairs. Any atoms interacting with each other while satisfying the criteria are considered to belong to the same molecule.

## 3. Results and Discussion

In this study, we perform three multiscale shock calculations along the different direction of HMX/HTPB composites. The time evolutions of system pressure, temperature, relative volume (V/V_0_) and pressure dependence of relative volume are displayed in Figure S1 in Supplementary Materials. The shock loading on the surface of (100) and (010) always cause the similar evolutions of pressure and relative volume, with final values of 50 and 0.54 GPa, respectively. While the system of the (001) surface has a higher pressure of 58 GPa, with corresponding relative volume of 0.52. Furthermore, the temperatures of the conditions of (100) and (001) almost reach the same values of 3150 K, while the (010) condition has a lower temperature of 2800 K. Furthermore, the pressure dependence of relative volume is compared with pure β-HMX (see Figure S1d). The HMX(100)/HTPB composite has more pressure sensitivity, while the other two conditions possess less sensitivity, compared with the pure HMX system, which reveals the anisotropic response properties of the different HMX/HTPB composites under shock loading.

### 3.1. The Interaction between HMX and HTPB with System Compression

The main interactions between HMX and HTPB molecules are proposed as hydrogen-bonding between hydroxyl-H, alkyl-H of HTPB and nitro-O of HMX [12,16]. However, as the system compresses, the intermolecular distances are significantly reduced, directly enhancing the interaction of adjacent molecules. Table S1 in Supplementary Materials shows the time evolutions of binding energy (defined as Formula (1) in Supplementary Materials) with different HMX/HTPB composites, which reveals that all the binding energies possess increments with initial shock loading. As compression continues, clearly repulsive interactions between these molecules are observed caused by space steric hindrance with large negative values of binding energy. 

**Figure 2:**
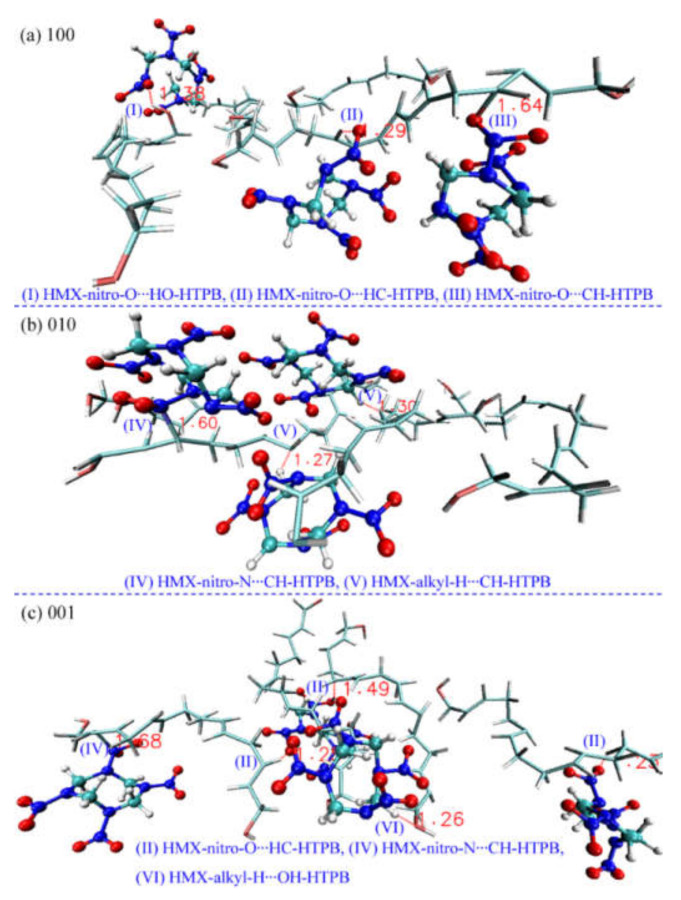
Mainly interaction patterns between HMX and HTPB molecules under shock loading.

Figure 2 displays the main interaction patterns of HMX and HTPB molecules with system compression. There are six typical interactions between different functional groups: (I) hydrogen bonding interaction between nitro-O and hydroxyl-H, (II) hydrogen bonding interaction between nitro-O and alkyl-H of HTPB, (III) interaction between nitro-O and alkyl-C of HTPB, (IV) interaction between nitro-N and alkyl-C of HTPB, (V) interaction between alkyl-H of HMX and alkyl-C of HTPB, and (VI) hydrogen bonding interaction between alkyl-H of HMX and hydroxyl-O. The three kinds of hydrogen bonding interactions are already detected in the thermal decomposition of HMX/HTPB composite [16]. The other interactions mainly derive from the drastic molecular deformation caused by shock compression, which would bring some new reaction pathways for HMX/HTPB decomposition, such as fast oxidation of alkyl-C with HTPB through the interaction of (III) and the aggregation of molecular chains by the interaction of (IV).

### 3.2. The Evolution of Electronic Properties under Shock Loading 

**Figure 3:**
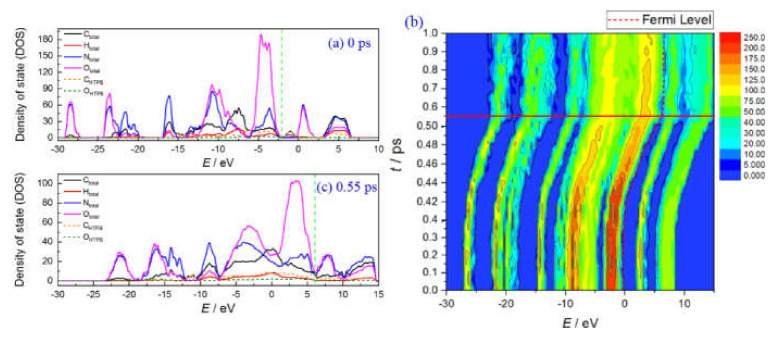
Density of states (DOS) for HMX(100)/HTPB composite: (**a**) at 0 ps, (**b**) DOS evolution, (**c**) at 0.55 ps.

From the previous study, we obtain the electronic properties of a pure HMX crystal under shock loading, and give the intrinsic mechanism of reaction initiation induced by electron excitation [37]. Here, we take HMX(100)/HTPB as an example to further study the electron structure evolution of the composite explosives. Figure 3 shows the time dependence of electronic density of states (DOS) for the HMX(100)/HTPB composite. The Fermi level is marked with a dotted line. The frontier band gap can be determined by the energy difference of the highest occupation molecular orbital (HOMO) and lowest unoccupied molecular orbital (LUMO). The initial DOS consists of several separated peaks with an energy gap of ~2.6 eV. Few impurity states are located in the forbidden band area, which mainly derive from the states of HTPB-C and HTPT-O (see Figure 3a). As volume compression, all the DOS distributions shift to the higher energy level, with the characteristic peaks becoming more and more dispersed (see Figure 3b). The frontier band gap decreases correspondingly. Up to 0.55 ps, the system realizes the metallization with a pressure of 52 GPa, which is comparable with that of the pure HMX system [37]. During this process, the impurity states derived from HTPB-C and HTPB-O have no significant changes, while the states of nitro-O and N from HMX crossing the Fermi level are the primarily factors for composite explosives metallization (see Figure 3c). The HTPB molecule does not affect the electronic properties of HMX at the initial shock compression stage, which also implies that the early decomposition of HMX remain the same.

### 3.3. The Population Evolutions of Main Chemical Bonds

**Figure 4:**
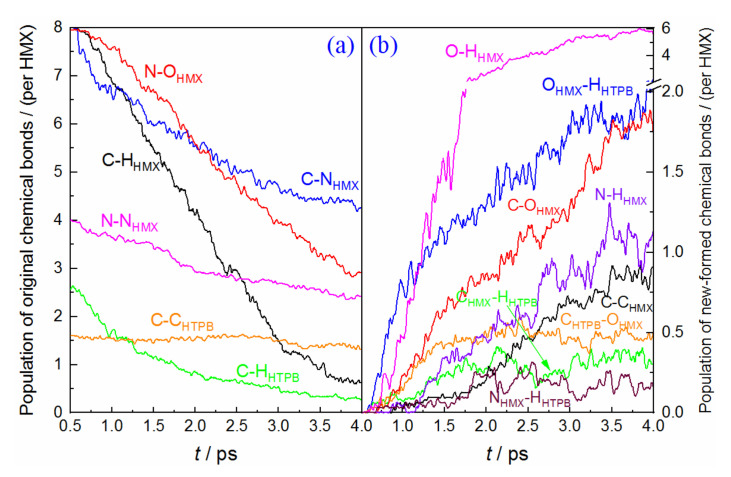
Time dependence of the main chemical bonds involved in HMX(100)/HTPB shock decomposition, (**a**) original chemical bonds, (**b**) new-formed bonds.

In this section, we investigate the time dependence of the populations of original and newly formed chemical bonds, which are normalized according to the number of HMX molecules (32 for (100), 36 for (010) and (001) conditions). Figure 4 displays the condition of the HMX(100)/HTPB composite. At the earliest stage, the population of the C−N bond has a fast decrease, with a decrement of 40, which indicates that the initial decomposition of HMX is mainly initiated by molecular ring rupture. However, the final C−N bond still keeps a considerable value, which represents that the high pressure would impede the deep fission of the molecular chain. The C−H bond starts to break after a slight delay, with a large number of O−H bonds generated, which denotes that the hydrogen transfer is also the primary pathway for the early stage. The dissociation of the N−O bond closely follows the breaking of O−H, indicating a delicate relationship between them. As for the N−N bond, its population decreases slowly, with more than 60% of the N−N bond remaining after simulation. For comparison, 75% of the N−N bonds are broken during HMX/HTPB thermal decomposition [16]. This apparently illustrates that the high pressure from shock compression efficiently inhibited the reaction activity of N−N fission [38,39]. As for the HTPB molecules, the population of the C−H bond displays a rapid decrease, for the fission ratio, of 41.25%, which is much greater than that of HMX (18.12%) from 0.5 to 1.2 ps. The corresponding O−H formation from HTPB also displays an advantage (see Figure 4b), which denotes that the C−H bond of HTPB has higher reaction activity than that within HMX at the early stage. Meanwhile, the oxidation of carbon atoms from the HMX and HTPB shows similar trends, with oxidation ratios of 8.59% and 17.86%, respectively. This also represents a higher reaction activity for HTPB-C. Although many O atoms combine with the original C skeletons to form the carbonyl or hydroxyl (see the configurations of HTPB chains at 4 ps shown in Figure S2a), no significant breaking of the C−C bond is observed. Their populations almost keep constant during this study. Furthermore, there is a considerable amount of newly formed N−H_HTPB_ and C−H_HTPB_ bonds deriving from the hydrogen transfer from HTPB to HMX. This phenomenon is also observed in thermal decomposition [16], but is apparently more intense under shock loading. At the end of the simulation, a large number of new C−C bonds are produced, which indicates that high pressure can efficiently promote C-fragment aggregation [40]. 

**Figure 5:**
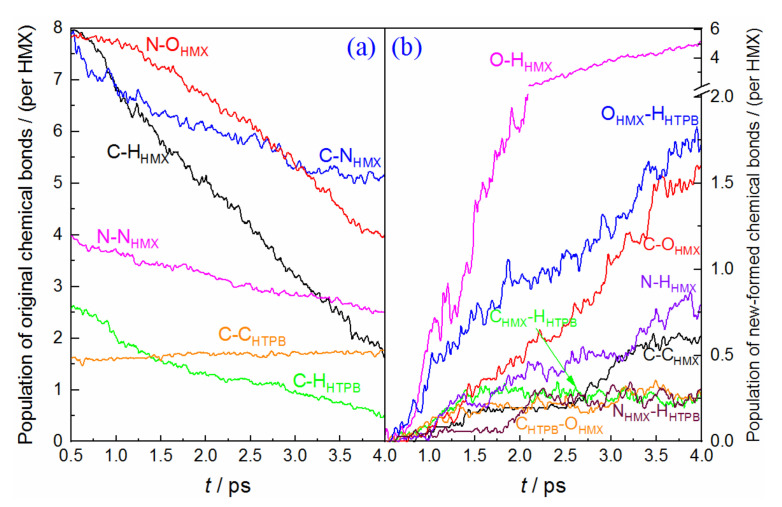
Time dependence of the main chemical bonds involved in HMX(010)/HTPB shock decomposition, (**a**) original chemical bonds, (**b**) new-formed bonds.

Figure 5 displays the evolution of the main chemical bonds in HMX(010)/HTPB decomposition. We can see that the primary trends for all the bonds are almost similar to the condition of HMX(100)/HTPB, but their reaction degrees are weaker than the latter. In the initial stage, about 35 C−N bonds rapidly rupture, followed by a fast breaking of the C−H bond. This indicates that the decomposition is also initiated by molecular ring dissociation and hydrogen transfer. The breaking of the N−O bond is much slower at the first 1.2 ps, and the formation of O−H has a slight corresponding delay. The oxidations of C and H within HTPB still have some advantages, but it is not as prominent as the condition of HMX(100)/HTPB.

**Figure 6:**
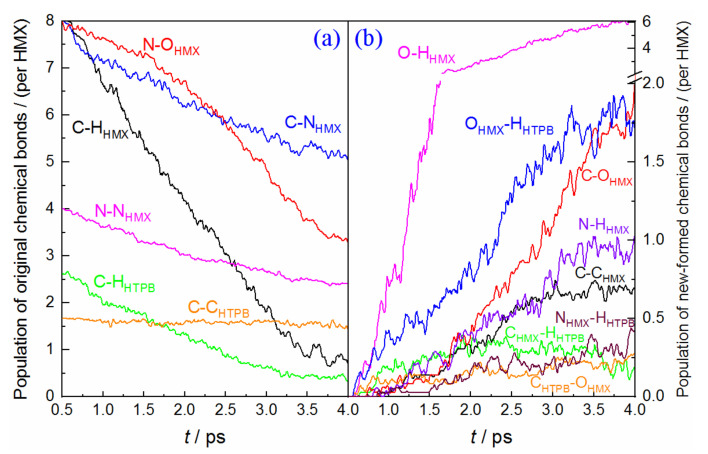
Time dependence of the main chemical bonds involved in HMX(001)/HTPB shock decomposition, (**a**) original chemical bonds, (**b**) new-formed bonds.

As to the condition of HMX(001)/HTPB (see Figure 6), only a few C−N bonds (~18) are observed to break at the initial decomposition, while the population of C−H bonds displays a drastic decrease, with a large amount of O−H bond formations. This illustrates that the dissociation reaction is mainly initiated by dehydrogenation oxidation, which is different from the conditions of HMX(100)/HTPB and HMX(010)/HTPB. Furthermore, the priority for HTPB-H oxidation is not considerable any more. The C oxidations both for HMX and HTPB are slower than that of HMX(100)/HTPB. Therefore, the effect of the HTPB molecule on the early dissociation of HMX is not as effective as the other two composites. However, more N−H_HTPB_ and C−H_HTPB_ bonds are produced during the following reaction, which denotes a stronger hydrogen transfer from HTPB to HMX.

### 3.4. The Concentration Evolutions of Main Species

**Figure 7:**
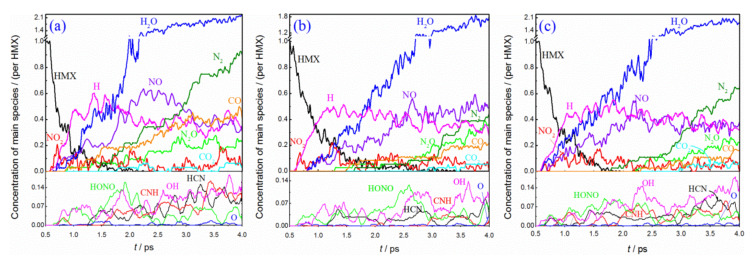
Main species involved in decomposition of HMX/HTPB composites: (**a**) HMX(100)/HTPB, (**b**) HMX(010)/HTPB, (**c**) HMX(001)/HTPB.

Figure 7 displays the time dependence of decomposition products involved in HMX/HTPB composites. The primary species of different HMX/HTPB composites almost keep consistent with each other, which consist of gaseous products of NO_2_, H_2_O, NO, CO, N_2_O, CO_2_, HCN, HONO and active radicals of H, OH, O, CNH. As shown in Figure 7a, NO_2_ is the first gaseous product of HMX(100)/HTPB, deriving from N-NO_2_ bond fission. Then, many H radicals are produced with C−H breaking. Although many O−H bonds are formed (see Figure 4), no corresponding OH or H_2_O are generated at the same time. Namely, this reaction process mainly consists of dehydrogenation and hydrogen transfer to nitro-O to form a hydroxyl. After that, NO and H_2_O are produced simultaneously, accompanying with the fast depletion of NO_2_. This illustrates that NO_2_ reduction with hydrogenation is an important pathway for H_2_O formation, which is consistent with the results obtained in pure HMX decomposition [29]. Furthermore, some CO, N_2_O, HCN and CNH are produced, with deep fission of the molecular ring. The N_2_ molecule is first formed at ~1.4 ps, and its concentration rapidly increases to reach ~0.9 at the end of the simulation. As for HMX(010)/HTPB composites (see Figure 7b), the main reaction trend is similar to the first one, but all the reactions are weaker than that of the former. For example, the first N_2_ is produced at ~1.6 ps, with a final concentration of ~0.4. Different from the two former conditions, H radicals and H_2_O molecules are first produced for the HMX(001)/HTPB composite (see Figure 7c), indicating that the hydrogen transfer oxidation, and N−O bond breaking are the important reaction pathways at the earliest stage. Then, NO_2_ and NO are generated in turn, and few OH and HONO molecules are also formed. The first N_2_ molecule is produced at ~1.4 ps, with its final concentration of ~0.6. In general, compared with thermal decomposition [16], the number of HCN and CNH fragments are smaller, while the formation of N_2_ is more favorable under shock loading. This can be mainly ascribed to the high pressure caused by the shockwave, which can significantly impede the breaking of C−N and N−N bonds (see Figure 4). This is beneficial for the formation of the molecular chain and N_2_ molecules. 

**Figure 8:**
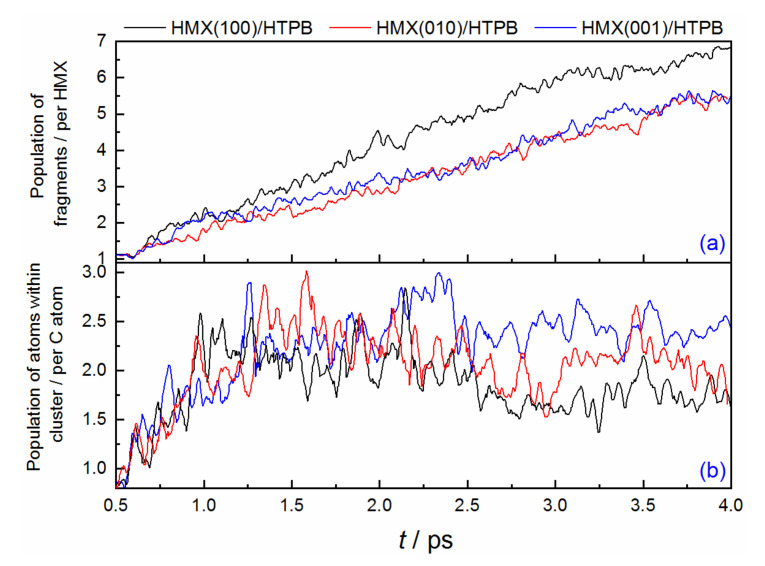
Populations for total fragments (**a**) and atoms in cluster (**b**) involved in different explosives composites.

Figure 8a displays the population evolutions of all the fragments. During the reaction process, the HMX(100)/HTPB composite has the smallest fragments versus the others, which indicates that the reaction activity is highest when there is shock loading on (100) surface. The other two show the similar trends for population changes, and the HMX(010)/HTPB displays a slightly weaker reaction performance. In addition to the small fragments, some big molecular chains or clusters are also formed. Figure 8b shows the atom population evolution of the clusters. The initial values of different composites are the same as each other, which derives from the HTPB molecular chains. As the reaction proceeds, many small fragments can aggregate together based on the original HTPB-C backbones, and they can further grow to form bigger heteratomic clusters [32] (see Figure S2 in Supplementary Materials). Among them, the cluster in the HMX(100)/HTPB system is smaller than the others, which is mainly because the fast dissociation to form a large number of active fragments within the system of (100) is beneficial for the reaction to small products. The (010) and (001) composites have comparable clusters, and the latter displays a slight advantage, which agrees with the result of the small fragments.

### 3.5. The Effect of Cluster Evolution on the Reaction Activity

**Figure 9:**
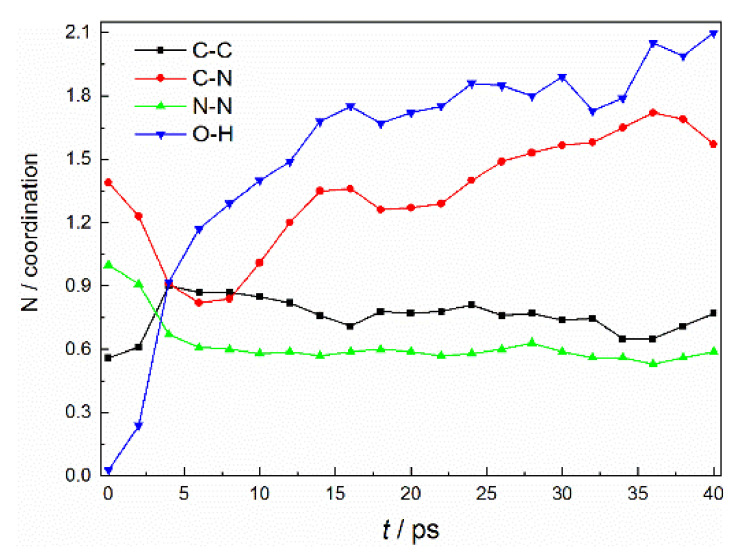
Amount of first coordination numbers of different atom pairs in the HMX(100)/HTPB system.

To uncover the effect of fragment aggregation in the following reaction, we investigate the microscopic structure features of the reaction system. Here, taking the HMX(100)/HTPB composite as an example, the MD simulation is extended to 40 ps. The radial pair distribution function (RDF) is employed to characterize the local structure orders of the product system, which is analyzed every two picoseconds (see Figure S3 in Supplementary Materials). The number of coordination (NC) of different atom pairs is determined by integration of RDF to the first coordination layer, which is shown in Figure 9. The initial coordination number for C−C comes from the HTPB chains. As the reaction proceeds, many new C−C bonds are produced (see Figure 4), and the corresponding NC rapidly increases within the first 4 ps. No significant change is observed for the C−C coordination number during the following process, mainly because a huge number of heteroatoms doped in a cluster impede the aggregation of C atoms (see Figure S4b in Supplementary Materials). The NC of the C−N pair decreases fast during the first 6 ps, indicating a drastic fission of C−N bonds in the HMX molecules. However, as the fragments collide, an increase in NC is revealed, which displays a more apparent increment than that of the C−C pair, which also illustrates that the C−N bond is more readily formed during the initial cluster aggregation, resulting in many C−N heteroatomic ring formations [32]. The initial NC of the N−N pair derives from the group of N−NO_2_ in HMX. As the N−N bond breaks to form NO_2_ and NO, the NC of N−N has a large decrement during the first 6 ps. After that, the remaining N−N coordination structures mainly exist as N_2_-stable molecules or -N−N- fragments implanted into newly formed clusters (see Figure S4b in Supplementary Materials); its NC almost keeps constant to the end of the simulation. The fast increase in NC for the O−H pair directly derives from alkyl-H oxidation. After the simulation, its NC almost reaches 2 and keeps constant, which denotes that most of the H and O atoms combine to form H_2_O molecules. The initially formed H_2_O molecules prefer to aggregate into small polymers, similar to the structures reported in a previous theoretical study [29], which has special catalytic and oxidative reaction activity, and which can efficiently promote the deep cleavage of HMX in the progression of the following reaction. However, after further aggregation, most of the H_2_O molecules coagulate together to form the quasi-water phase, and they depart from the heteroatom cluster (see Figure S4c in Supplementary Materials). As a result, the initial reaction activity is diminished. Overall, the coordination numbers of C significantly increase as the reaction proceeds, especially for the C−N pairs. Some of the formed C−N heteroatomic rings possess high stability, which can stably exist in the system for several picoseconds as revealed in a previous work [30]. Many active C and N groups are locked in the cluster, which not only delay N_2_ formation and liberation, but also impede the purification of C cluster, resulting in a slower and weaker reaction process.

**Figure 10:**
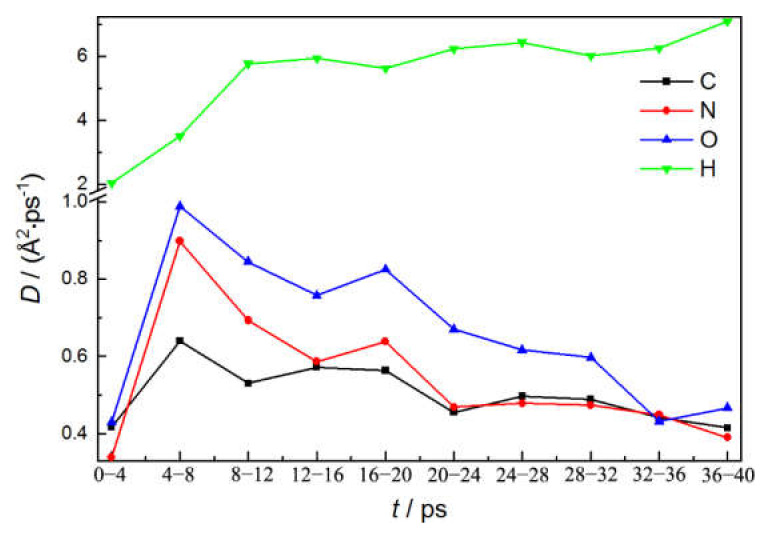
Diffusion coefficient of different atoms in HMX(100)HTPB shock decomposition.

The thermal motion and vibration of individual atoms are further analyzed to reveal their reaction activity evolutions accompanied with carbon clustering. A displacement rate (D) similar to the diffusion coefficient is employed, whose time dependence relationship is shown in Figure 10, which is determined by fitting the mean square displacement (MSD) of atoms based on MD trajectory [41,42] (the detailed illustration and typical MSD curves are displayed in Figure S5 in Supplementary Materials). The displacement rates of C, N, O atoms quickly increase during the first 4 ps and then gradually decrease. As decomposition is initiated, many HMX molecular structures are destroyed, with a large number of small fragments formed, which is that the newly formed fragments possess a much higher freedom of motion, which can result in fast diffusion of the atoms within them, which is beneficial for effective collision between each other, causing a fast reaction within the system. However, accompanying the larger cluster generated (see Figure 9), many atoms are bound within it and can only vibrate around their equilibrium positions. The corresponding motion is mainly caused by a slow evolution of the cluster’s dynamic structure. Therefore, the displacement rate of the reaction active groups is reduced by the formation of the cluster. The efficient collisions between them are correspondingly weakened, which could delay progression of the following reaction. In contrast, the H atoms have the highest displacement rate, and almost keep constant after 12 ps, which is mainly because the major H atoms convert into H_2_O molecules and are separated from the solid cluster. Their diffusion motion depends on themselves, which is beneficial for phase separation.

## 4. Conclusions

The anisotropic reaction properties for different HMX/HTPB composites under shock loading are investigated based on quantum-based molecular dynamics simulations. Some new interactions between HMX and HTPB molecules, such as nitro-O and alkyl-C of HTPB, nitro-N and alkyl-C of HTPB, alkyl-H of HMX and alkyl-C of HTPB, are discovered while subjected to shock compression. The metallization of the HMX/HTPB composite is mainly caused by electron excitation of nitro-O and N from HMX, with a critical pressure of 52 GPa. The shock decompositions of HMX(100)/HTPB and HMX(010)/ HTPB are mainly initiated by molecular ring breaking and alkyl hydrogen transfer. The initial oxidations of C and H within HTPB display advantages, resulting in a delay for that of HMX, while for the condition of HMX(001)/HTPB, the dissociation is primarily started with alkyl dehydrogenation oxidation, and a stronger hydrogen transfer from HTPB to HMX during the following reaction stage is detected. Compared with thermal decomposition, the formations of the N_2_ and C-N fragments are more favorable, which is mainly ascribed to the impediment of C-N and N-N bonds breaking under high pressure. Furthermore, the bonded structure and displacement rate of the reaction system are analyzed to uncover the negative effects of fragment aggregation on the progression of the following reaction.

## Data Availability

Not applicable.

## Supplementary Materials

The following supporting information can be downloaded at: https://www.mdpi.com/article/10.3390/molecules27092787/s1, Figure S1: time dependence of system pressure, temperature, volume, and pressure dependence of relative volume [38]; Definition of binding energy; Table S1: time dependence of binding energy; Figure S2: C-C chains structure from HTPB at 4 ps; Figure S3: RDF of different pairs; Figure S4: final product structure at 40 ps; Definition of displacement rates (D); Figure S5: time dependence of mean square displacement.

## Sample Availability

Samples of the compounds are available from the first author.
