# Supplementary material for: Anisotropic Reaction Properties for Different HMX/HTPB Composites: A Theoretical Study of Shock Decomposition
Source: Molecules. 2022 Apr 27;27(9):2787. doi: 10.3390/molecules27092787 (PMC9102234; doi:10.3390/molecules27092787)
Supplement: Supplementary file 1 [file molecules-27-02787-s001.zip › molecules-1657637-supplementary.pdf]

Supplementary Materials

Anisotropic Reaction Properties for Different HMX/HTPB Composites: A Theoretical Study of Shock Decomposition

Zheng-Hua He[1], Yao-Yao Huang[1], Guang-Fu Ji[1], Jun Chen[,2], Qiang Wu[*,1]*

[1]*National Key Laboratory of Shock Wave and Detonation Physics, Institute of Fluid Physics, China Academy of Engineering Physics, Mianyang 621900, Sichuan, China.*

[2]*National Key Laboratory of Computational Physics, Institute of Applied Physics and Computational Mathematics, Beijing 100088, China.*

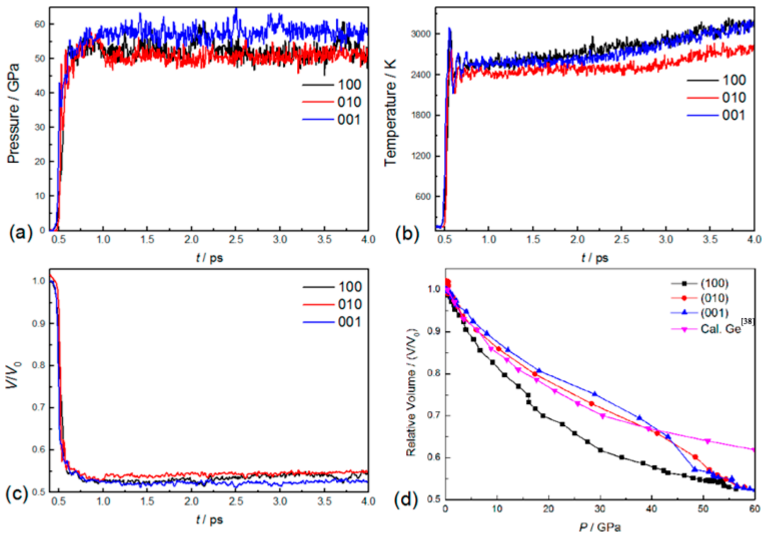

Figure S1 Time dependence of system pressure (a), temperature (b), relative volume (c) and pressure dependence of relative volume (d) of HMX/HTPB composites under shock loading. (ref 38 for theoretical calculation data of pure HMX.)

The binding energy is defined as the negative value of the energy difference between PBX composite and its components, as shown in formula (S1). Where E_{PBX} is the total energy for the PBX, E_{HMX} and E_{HTPB} represent the single-point energies of HMX and HTPB, respectively.

$$E_b = -E_{inter} = -(E_{PBX} - E_{HMX} - E_{HTPB}) \tag{S1}$$

Table S1 Time dependence of binding energy between HTPB and HMX for different composites.

	Binding Energy / kcal·mol^{-1}			
t / ps	0.4	0.45	0.5	0.55
100	69.86	73.42	79.41	-502.46
010	107.19	105.97	119.13	-484.88
001	78.94	304.57	-30.85	-1416.43

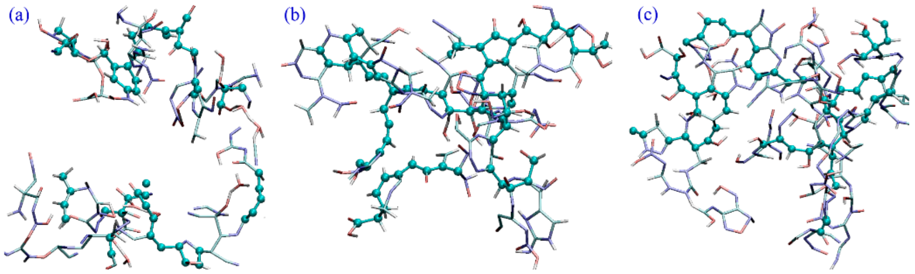

Figure S2 Final C-C chains from HTPB in different composites at 4 ps, (a) HMX(100)/HTPB, (b) HMX(010)/HTPB, (c) HMX(001)/HTPB.

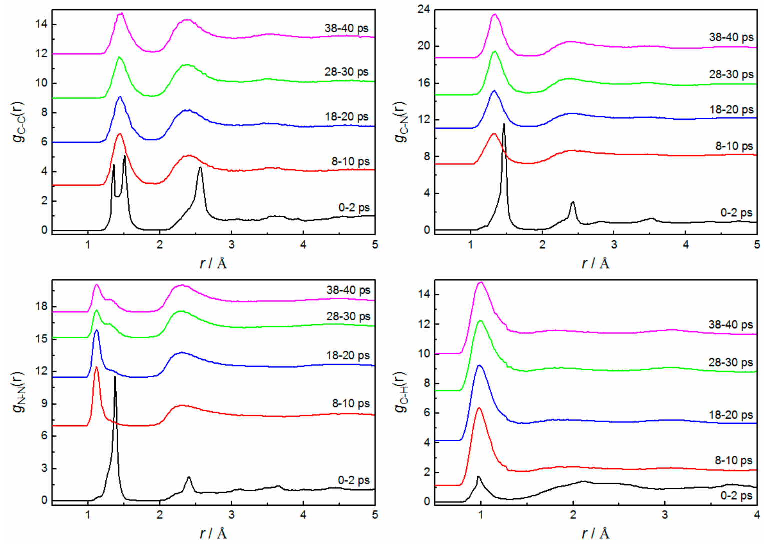

Figure S3 Radial pair distribution function (RDF) of different atomic pairs.

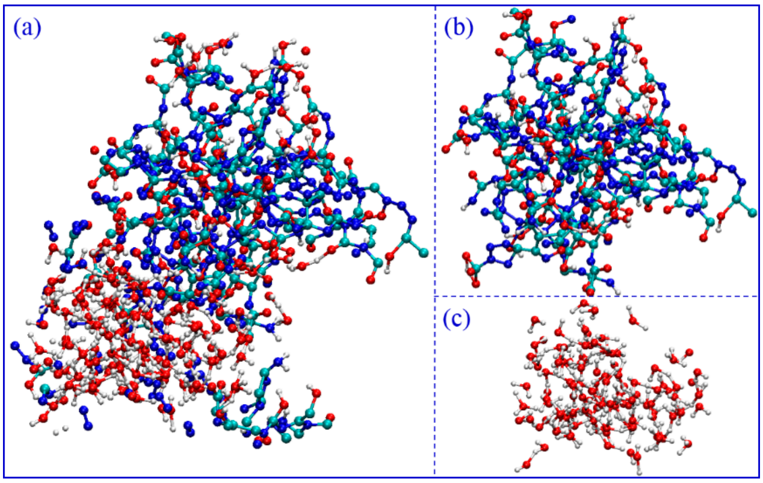

Figure S4 Snapshots for the final product structure of HMX(100)/HTPB composite, (a) whole system, (b) heteroatomic cluster, (c) water cluster.

The displacement rates (D) of different atoms are determined similar as diffusion coefficient by explore the mean square displacement (MSD), which can be defined as formula (S2). The slope for MSD-Δt curve is relevant with D, which can be obtained using the formula (S3). And some of the MSD versus time interval Δt are shown in Fig. S5.

$$MSD(\Delta t) = \frac{1}{T-\Delta t}\int_0^{T-\Delta t}\left[r(t-\Delta t)-r(t)\right]^2 dt = \left\langle \left[r(t-\Delta t)-r(t)\right]^2 \right\rangle \quad \text{(S2)}$$

$$D = \frac{1}{6}\lim_{\Delta t\to\infty}\frac{dMSD}{d\Delta t} \quad \text{(S3)}$$

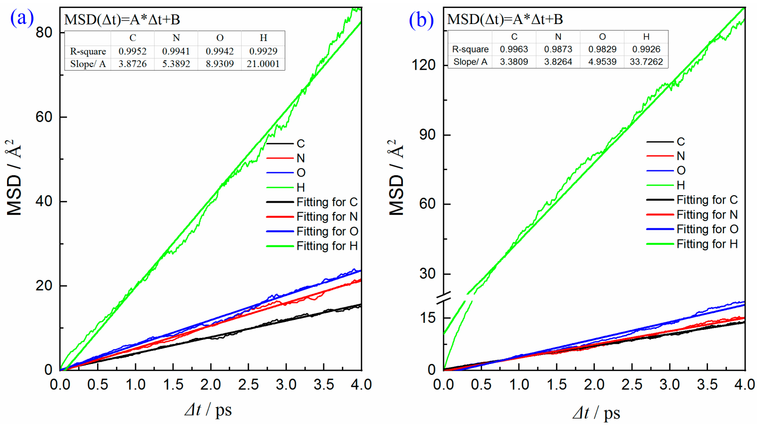

Figure S5 Mean square displacement of different atoms versus time interval, (a) for 4-8 ps, (b) for 16-20 ps.
